# Consumption of a Polyphenol-Rich Grape-Wine Extract Lowers Ambulatory Blood Pressure in Mildly Hypertensive Subjects

**DOI:** 10.3390/nu7053138

**Published:** 2015-04-30

**Authors:** Richard Draijer, Young de Graaf, Marieke Slettenaar, Eric de Groot, Chris I. Wright

**Affiliations:** 1 Unilever Research & Development Vlaardingen, Olivier van Noortlaan 120, Vlaardingen 3133 AT, The Netherlands; E-Mails: young-de.graaf@unilever.com (Y.G.); meyer.ma@menzis.nl (M.S.); ciwright26@hotmail.com (C.I.W.); 2 Academic Medical Center Amsterdam, Department of Vascular Medicine, Meibergdreef 9, Amsterdam 1105 AZ, The Netherlands; E-Mail: ericdg@xs4all.nl

**Keywords:** red wine, grape, polyphenols, blood pressure, vascular function, catechins, procyanidins

## Abstract

Polyphenols in grape and wine have been suggested to contribute to the cardiovascular health benefits of the Mediterranean lifestyle. The reported effects of grape products on blood pressure (BP) remain, however, equivocal. In a double-blind placebo controlled crossover study, the effect of two grape extracts on BP and vascular function was assessed in 60 untreated, mildly hypertensive subjects after four weeks intervention. Both extracts (grape-red wine and grape alone) had high concentrations of anthocyanins and flavonols, but the grape alone was relatively poor in catechins and procyanidins. Parameters measured included ambulatory and office BP, flow-mediated vasodilation, arterial distensibility, platelet function and plasma lipoproteins. Results showed that 24-hour ambulatory systolic/diastolic BPs were significantly lower in the grape-wine extract intervention (135.9 ± 1.3/84.7 ± 0.8 mmHg; mean ± SEM) compared to placebo (138.9 ± 1.3/86.6 ± 1.2 mmHg), predominantly during daytime. Plasma concentrations of the vasoconstrictor endothelin-1 decreased by 10%, but other measures of vascular function were not affected. Grape juice extract alone had no effect on BP or any measures of vascular function. Polyphenol-rich food products, and may be specifically catechins and procyanidins, may thus help sustain a healthy BP and contribute to the healthy Mediterranean lifestyle.

## 1. Introduction

The Mediterranean lifestyle has often been advocated as a way of living to ensure (cardiovascular) health with the consumption of red wine playing a central role [1]. Other alcohol containing beverages do not have this health benefit, which suggests that another main constituent of wine, not present in most other alcoholic beverages, may be responsible; polyphenols are a likely candidate as their consumption has been associated with health benefits [2]. Polyphenols are present in a wide range of food products, from fruit, tea, coffee and cocoa to olive oil. [3]. Based on epidemiological and interventional studies, convincing evidence relating polyphenol intake to cardiovascular health has been derived from cocoa and tea studies. The effects reported are a lowering of blood pressure (BP) and an improvement in endothelial function [4,5]). Results from clinical studies with grape products, including grape juice, grape seed and wine are more ambiguous [6,7,8,9,10,11,12]. In fact, in some cases, polyphenol dose or composition may have not been sufficient to exert a physiological effect. We therefore designed the present clinical study to answer the clinically relevant question “do grape-derived polyphenols lower BP?” To avoid the influence of alcohol, we used spray-dried grape and wine extracts. We aimed for a polyphenol dose of 800 mg per day, which is at the high end of normal dietary intake [13], ensuring that the dose would not be the limiting factor in the trial design. Furthermore, a mix of two grape products was composed, which contained all grape polyphenol classes. This mix consisted of a polyphenol-rich extract of grape juice extract (MegaNatural™; rich in anthocyanins) combined with a red wine extract (Provinols™; complex polyphenol composition, particularly rich in the polyphenol classes flavanols, flavonols, procyanidins, phenolic acids and stilbenes [14]). The grape juice extract alone was also tested, representing a grape product with a comparatively less complex polyphenol composition. The test extracts were administered to mildly hypertensive subjects. Ambulatory BP and office BP were measured. The effect of these extracts on vascular function also remains unclear, so the secondary objective was to assess their effects on endothelial function, as assessed by flow-mediated vasodilation (FMD), and local (arterial distensibility), regional (pulse wave form analysis; PWFA) and systemic (pulse wave transit time) arterial stiffness as well as plasma lipoproteins and platelet function. The urinary phenolic metabolites identified in the present study have been published previously [14].

## 2. Experimental Section

### 2.1. Recruitment and Screening

The study was run at Unilever R&D Vlaardingen and was approved by an independent external medical ethical committee at the University of Wageningen (the Netherlands) (d.d. 22 October 2004; study code 04/08-UHI 0412P). Volunteers were recruited using a subject database (1977 letters), and advertised by distributing leaflets in the local area (140,000 leaflets). The volunteers were screened and selected on the basis of their blood pressure values and other health criteria. The key inclusion criteria were: males and females, age ≥35 and ≤75 years, office SBP 130–179 mmHg and DBP ≤ 100 mmHg, BMI ≥ 18.0 and ≤ 32.0 kg/m^2^, apparently healthy (no reported current or previous metabolic diseases, chronic gastrointestinal disorders, cardiovascular or renal disease). Exclusion criteria were: use of medication interfering with the study parameters (anti-hypertensives, hormone replacement therapy and chronic use of NSAID’s), on a diet, smoking, having an irregular pulse or intensely sporting (>10 h/w). Of the 918 persons who expressed an interest in the trial, 728 attended an information meeting, and 240 people met the inclusion/exclusion criteria and were invited to a second screening visit, which included taking a 9 mL blood sample and a urine sample. Eighty-two people met all criteria, and from this 60 subjects with the highest systolic blood pressure (SBP) were enrolled in the trial. All participating subjects provided informed consent.

### 2.2. Study Design

Subjects were randomized in a double-blinded, incomplete-crossover design with two treatments and a placebo control treatment. All treatments were provided in red gelatin capsules, ensuring blinding of the study. Subjects were assigned to the following three treatments: placebo capsules; capsules with grape juice extract alone; and, capsules with a mixture of grape and wine extract. The study duration was 10-weeks. This started with a 2-week run-in period and two intervention periods, each lasting 4 weeks. During the run-in period the subjects consumed six placebo capsules per day, three at breakfast-time and three at dinner-time. The time capsules were ingested was similar during each of the intervention periods. The study was designed as an incomplete crossover trial. Hence during the run-in period all subjects consumed the placebo control (cellulose-filled capsules). Then during the subsequent intervention periods, 30 subjects consumed the grape-wine extract capsules (intervention group 1) or placebo in randomized order, whilst the other 30 subjects consumed the grape juice extract capsules (intervention group 2) or placebo in randomized order.

During the entire study period, subjects were asked to minimize changes in their habitual diet and to avoid alcohol, high salt-containing products, foods containing plant sterols and stanols, and to refrain from taking vitamins supplements. Twenty-four-hour urine was collected on the pre-test day. On the test day subjects were fasted and reported to Unilever R&D Vlaardingen’s test center where office BP was measured and blood samples were taken. Subjects were then allowed to consume a light breakfast with 3 capsules (placebo control, intervention 1 or intervention 2). Office BP, FMD, arterial distensibility, pulse wave transit time, and PWFA were measured between 90 and 180 min after breakfast was consumed, focusing particularly on acute-on-chronic effects. After a second blood sample was taken, 24-h ambulatory BP was measured.

### 2.3. Measurements

All measurements were done at baseline (day 0; end of the run-in period) and at the end of the intervention periods (after 4 weeks).

#### 2.3.1. Ambulatory BP

Ambulatory BP was monitored for 24 h at the non-dominant arm by Spacelabs monitor type 90217, according to the manufacturer’s directions. The monitor was mounted to the arm immediately and programmed to take readings every 20 min during the day, starting immediately after the vascular measurements and then every 60 min during the night. Subjects received instructions as to the operation of the monitor, and were asked to refrain from any strenuous activity during the measurement period. They were also asked to sit down, if possible, or stay still, and relax their arm during readings. Apart from this no other restrictions were placed on the subjects, and they were asked to carry on with their day as normal. Subjects were asked to keep a diary during this period to capture the time they woke up, went to sleep, ate and conducted any particular activity whilst measurements were being made.

#### 2.3.2. Office BP

Office BP and pulse rate were measured after 15 min of rest with an automatic arm cuff method using Omron BP monitors. Subjects were sitting and the pressure cuff was positioned on the non-dominant arm at heart level. Each measurement comprised three successive measurements conducted within a 10 min period. The mean of the last two was calculated and used in this study. The subjects were blinded whilst all BP measurements were taken and were blinded to the BP results during the entire study.

#### 2.3.3. Flow-Mediated Vasodilation (FMD)

Subjects were supine whilst recordings of FMD were conducted using ESAOTE AU5 echo-Doppler equipment with automatic wall tracking software [6]. The subject’s right arm was comfortably immobilized in the extended position to allow consistent access to the brachial artery for imaging. The brachial artery was imaged in the upper arm (about 5 cm above the antecubital crease) in B-mode. Baseline brachial artery diameter was recorded at least three times before measurements of FMD, pulse wave transit time and arterial distensibility were started.

The FMD protocol was initiated by inflating a cuff around the right forearm to 250 mmHg and this was used to occlude the artery for 5 min. After 5 min the cuff was deflated and the arterial diameter was measured for a further 5 min. During measurements of the arterial diameter, subjects were asked to refrain from moving and talking. FMD was defined as the maximal arterial diameter within the 5-min period following cuff deflation. This change was then expressed as a percentage change using the following calculation: (maximal change in arterial diameter from baseline/baseline arterial diameter) × 100. The time to reach the maximum diameter was automatically calculated and reported as FMD time.

#### 2.3.4. Systemic Arterial Distensibility

A 3-lead ECG was used to record the electrical activity of the heart. The echo equipment was used to calculate brachial pulse wave transit time (from the heart-to-brachial artery) from the difference between the R-wave, reflecting ventricular systole, and the peak of the brachial artery pressure wave recorded by using an echo-transducer. Carotid pulse wave transit time (from the heart-to-carotid artery) was also measured using a similar approach but using the peak carotid artery pressure wave. This was automatically measured after the FMD measurement.

#### 2.3.5. Local Arterial Distensibility

Local arterial distensibility was assessed from the distensibility coefficient (DC) calculated using the equation DC = (∆A/A)/∆P = 2 × ∆D/D/∆P. A is the diastolic cross-sectional area; ∆A is the change in cross-sectional area; ∆P is the local pulse pressure (*i.e*., SBP-DBP); D is the diastolic diameter; and, ∆D is change in diameter during the heart cycle [15]. The arterial diameter was measured using the ultrasound equipment. BP was derived from a BP measurement, which was recorded after the FMD measurement using a calibrated Omron device (see office BP, but in this case in the supine position).

#### 2.3.6. Regional Arterial Distensibility

Pulse waveform analysis (PWFA) was assessed after the FMD measurement using a radial tonometer (HD/PulseWave™ CR-2000 CardioVascular Profiler; Hypertension Diagnostics™, USA). Prior to the PWFA, blood pressure was taken by a cuff placed around the non-dominant arm. PWFA includes acquisition of calibrated radial artery BP waveform data. Non-invasive radial artery waveforms were recorded for 30-s by placing a sensor on the radial artery near the wrist of the dominant arm. Each measurement comprised of two consecutive measurements, which were repeated (4 measurements in total). To obtain a measure of arterial compliance (absolute change in area for a given change in pressure), a model was used that divides the total systemic arterial compliance into large and small arterial compliance. Large arterial compliance reflects the capacitive compliance in the large conduit arteries, which stores blood during systole, and small arterial compliance reflects compliance of smaller resistance arteries. These parameters were automatically calculated by the HD/PulseWave™ CR-2000 CardioVascular Profiler.

#### 2.3.7. Platelet Aggregation

Platelet aggregation was measured in the fasted state and then 150 min after the last capsule was ingested. Platelet aggregation was determined in citrated whole blood using the Platelet Function Analyser (PFA-100^®^ system, Siemens, USA). In the PFA-100 measurements, collagen either combined with adenosine-diphosphate (ADP) or epinephrine cartridges was used. Blood was collected from subjects using vacutainers with 3.2% citrate. Within 30 min, sampled blood was inserted into the PFA-100 and the aggregation time determined.

#### 2.3.8. Polyphenols and Polyphenol Metabolites

Urinary polyphenols and polyphenol metabolites identification was done using NMR- and GC-MS profiling techniques. Methodology and results of the current study have been reported previously by Van Dorsten *et al*. [14]).

#### 2.3.9. Plasma Markers

Total cholesterol, HDL-C, LDL-C and triglycerides were measured in serum samples on a quantitative clinical chemical analyzer (Hitachi 912, Japan). Plasma endothelin-1 (ET-1) was measured by an ELISA Kit (R & D Systems, USA).

### 2.4. Test Products

The wine and grape juice extract mix comprised of approximately two parts red wine extract (Provinols™; Seppic, France) and one part Rubired grape juice extract (MegaNatural™; Polyphenolics, USA). Both test products contained 800 mg of polyphenols, based on gallic acid equivalents. This equates to roughly 5 glasses of red wine. The polyphenolic content was measured using Folin-Ciocalteu analysis and summarized as follows:

- placebo: capsules containing microcrystalline cellulose (Avicel PH101, FMC Biopolymer);
- grape-wine extract: 800 mg polyphenols of which 550 mg polyphenols derived from Provinols™, and 250 mg of MegaNatural™ Rubired grape juice extract; and
- grape juice extract: 800 mg polyphenols of MegaNatural™ Rubired grape juice extract.

The grape juice and wine extracts were analyzed for nutritional values by the Institute Kuhlmann in Germany and total polyphenol content was analyzed by Unilever R & D using ^1^H-NMR technique.

The grape juice extract contained 83 g polyphenols, 6.5 g water, 5 g sugars, 2.3 g protein, 1 g fiber, <0.1 g fat, and 2 g ash per 100 g extract. The wine extract contained 81 g polyphenols, 6.5 g water, 0.4 g sugars, 7.9 g protein, 1 g fiber, <0.1 g fat, and 1 g ash per 100 g extract [14].

The capsules filled with the extracts were produced by Well Plus Trade in Germany. The grape extract(s) were mixed with cellulose (Avicel PH 101) to obtain a volume sufficient to fill six test capsules each with a volume of 0.67 mL (Capsugel Conisnap capsules no. 0).

A questionnaire was used to check that the test product was being taken as instructed. Each day subjects reported when the test product was consumed and at what time in the morning/evening it was consumed. Seventy percent of subjects fully complied with the instructions for consumption. The remaining 30% of subjects were 94 to 99% compliant.

## 3. Statistical Analysis

Based on a within-subject variation of 44 (a number derived from previous internal studies), a power of 0.8 and alpha of 0.05, we calculated that 27 subjects would be required to pick up a SBP reduction of 5 mmHg in a cross-over design. Taking a number of dropouts into account and for logistical reasons, 30 subjects were included per extract intervention. Prior to statistical analyses and de-blinding of the study, a blind review of the data was performed. The objective of the blind review was to identify subjects and/or data points that should be excluded from statistical analysis based on events that might have affected data validity such as compliance, consumption of restricted foods, and adverse events, such as illness, use of medication, body weight changes, aberrant clinical chemistry/hematology and laboratory error. Possible outliers in datasets were detected by probability plots. Decisions on exclusion of a person or data points were made based on the period in which the event occurred (relation to sampling dates), the parameters that could have been affected by the event, correlation with other aberrant observations and scientific reasons. Based on these criteria a limited number of data was excluded (e.g., four 24-h ambulatory BP measurements from a total of 120 measurements in the study, and none of the office BP measurements). Some vascular data were excluded due to technical problems and low quality of the obtained data. No data were excluded due to background diet, lifestyle or compliance criteria. Then, de-blinding of the treatments was done by a staff member, who was not personally involved in the clinical study. Data were analyzed using a two-way mixed model ANOVA (Proc Mixed, SAS software V9.1) with treatment, period and their interaction as model terms, and including the baseline as adjusting variable and subject as random factor to compare the absolute differences after the intervention periods between the two extract treatments and the placebo. A *p*-value of less than 0.05 was considered statistically significant. Descriptive statistics are presented as mean and standard deviation (SD). Values reported in the text and tables are Least-Squares Means (LSMeans) and standard error of the mean (SEM).

## 4. Results

Sixty subjects started this study. During the run-in period five subjects dropped out due to medical reasons (*n* = 4) or because of participation in another trial (*n* = 1). These five subjects were replaced to ensure 60 subjects completed the study. The final study population consisted of 33 males and 27 females. The average age was 57.6 ± 9.9 years, BMI was 26.3 ± 4.1 kg·m^−2^ and BP was mildly elevated (office SBP/DBP were 139.9 ± 12.2/84.8 ± 8.3 mmHg, respectively).

Thirty-four adverse events (AEs) were reported during the run-in period, 56 during placebo intervention, 13 during the grape intervention and 11 during the grape-wine intervention. AEs were not judged to be causally related to the test products. The most commonly reported AEs were common cold (acute nasopharyngitis), headache, influenza and dental caries. No serious AEs were reported (see supplementary research data, Table S1).

**Figure 1:**
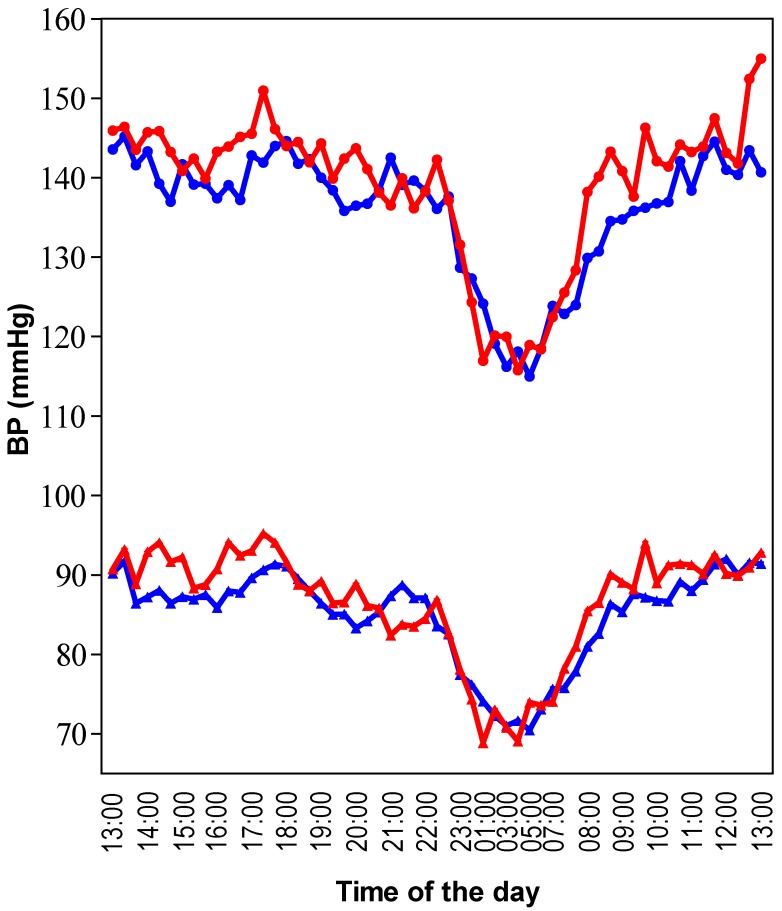
Twenty-four-hour ambulatory recordings of SBP and DBP. This data was extracted from intervention group 1 where placebo was compared with the grape-wine extract. Mean data per time point from 30 subjects is presented. The red line represents placebo and the blue line the grape-wine extract. Circles represent SBP and triangles DBP. Time zero started at 1:00 p.m. and ended at 1:00 p.m. on the following day. Compared to placebo, SBP and DBP were lower during the daytime with the grape-wine extract, but the extract did not affect the nighttime BP change.

Twenty-four-hours ambulatory BP was significantly lower after consumption of the grape-wine extract compared to placebo; SBP and DBP decreased by 3 and 2 mmHg, respectively. The effect was exclusively observed during the daytime, either defined by a predefined daytime or the actual awake period (Figure 1; Table 1). Heart rate did not significantly change. No significant effects on BP were found for the grape juice alone.

Although mean office BP values were lower in the grape-wine intervention compared to placebo on four different measurement occasions, the difference did not reach significance on any occasion. Office BP difference were in the fasted state (SBP/DBP −2.4/−1.1 mmHg), after breakfast (−1.7/−1.0 mmHg), after the FMD procedure (−1.3/−2.0 mmHg), and after the PWFA procedure (−2.4/−1.7 mmHg), respectively. Office BP was also not affected by the grape juice alone on a number of measurement occasions during the test day (SBP changed by between −0.7 and +2.1 mmHg; DBP between −0.4 and +1.4 mmHg).

Vascular function parameters were extensively evaluated (Table 2). Endothelial function assessed by FMD was similar in both intervention groups and there was no indication of endothelial dysfunction with values around 5% [16]. FMD response time was not affected. Propagation time of the pulse wave, an indicator of systemic vascular distensibility, from either heart to brachial artery or from heart to carotid artery, was not significantly changed by either test intervention. Indicators of local vascular distensibility were also not affected by test interventions.

**Table 1:** The effect of grape-wine extract and grape extract on BP and heart rate (HR) parameters.

Parameter	Units	Intervention group 1 (*n* = 28)		Intervention group 2 (*n* = 29)	
		Placebo	Grape-wine	Placebo	Grape
24 h SBP	mmHg	138.9 ± 1.3	135.9 ± 1.3 *	132.1 ± 1.4	131.8 ± 1.3
Daytime fixed SBP	mmHg	143.9 ± 1.6	140.0 ± 1.6 ^#^	136.8 ± 1.5	135.9 ± 1.5
Daytime diary SBP	mmHg	142.0 ± 1.4	138.3 ± 1.4 *	135.5 ± 1.6	135.7 ± 1.6
Night-time fixed SBP	mmHg	118.9 ± 1.6	119.6 ± 1.6	115.7 ± 1.8	114.1 ± 1.8
Night-time diary SBP	mmHg	119.7 ± 1.5	118.7 ± 1.5	116.0 ± 1.7	114.6 ± 1.6
24 h DBP	mmHg	86.6 ± 1.2	84.7 ± 0.8 *	79.6 ± 0.6	79.0 ± 0.6
Daytime fixed DBP	mmHg	90.0 ± 1.1	87.5 ± 1.1 **	82.8 ± 0.7	81.9 ± 0.7
Daytime diary DBP	mmHg	88.3 ± 0.9	86.2 ± 0.9 **	81.4 ± 0.7	80.8 ± 0.7
Night-time fixed DBP	mmHg	71.5 ± 1.0	72.6 ± 1.0	67.5 ± 1.0	65.9 ± 1.0
Night-time diary DBP	mmHg	71.9 ± 1.0	72.1 ± 1.0	68.6 ± 0.9	67.0 ± 0.9
24 h HR	bpm	73.8 ± 1.2	75.7 ± 1.2	72.0 ± 0.9	71.4 ± 0.8
Daytime fixed HR	bpm	77.4 ± 1.8	79.2 ± 1.8	75.8 ± 1.4	75.4 ± 1.5
Daytime diary HR	bpm	75.0 ± 1.7	76.1 ± 1.7	74.2 ± 1.4	73.2 ± 1.4
Night-time fixed HR	bpm	61.8 ± 1.2	62.0 ± 1.3	59.1 ± 1.2	58.8 ± 1.1
Night-time diary HR	bpm	61.6 ± 1.3	61.5 ± 1.3	59.1 ± 1.2	58.9 ± 1.2

Data is presented as mean ± S.E.M. Daytime diary: BP and HR were measured whilst the subject was awake; daytime fixed: BP and HR were measured from 10:00 am to 8:00 pm. Night-time diary: BP and HR were measured whilst the subject was asleep; night-time fixed: BP and HR were measured from 0:00 a.m. to 6:00 a.m. * *p* < 0.05, ^#^ *p* < 0.01, ** *p* < 0.005 *versus* placebo.

**Table 2:** The effect of grape-wine extract and grape extract on systemic, regional and local vessel stiffness.

Parameter	Units	Intervention group 1 (*n* = 26)		Intervention group 2 (*n* = 26)	
		Placebo	Grape-wine	Placebo	Grape
Brachial artery diameter	mm	4.23 ± 0.02	4.22 ± 0.02	4.29 ± 0.03	4.33 ± 0.03
Brachial FMD	mm	4.43 ± 0.01	4.42 ± 0.01	4.49 ± 0.02	4.53 ± 0.02
Brachial FMD	%	4.8 ± 0.3	4.9 ± 0.3	5.0 ± 0.5	4.9 ± 0.5
Brachial FMD time	s	47.1 ± 0.2	43.8 ± 0.2	56.0 ± 0.4	51.1 ± 0.3
PT heart-brachial artery	ms	122.8 ± 1.1	124.7 ± 1.2	130.6 ± 1.2	127.5 ± 1.2
PT heart-carotid artery	ms	87.6 ± 1.3	86.3 ± 1.5	92.9 ± 1.3	91.3 ± 1.2
DC carotid artery	MPa^−^^1^	29.7 ± 1.1	26.9 ± 1.2	28.6 ± 1.5	27.8 ± 1.4
Small arterial compliance	mL/mmHg × 100	5.2 ± 0.2	5.1 ± 0.2	4.5 ± 0.2	4.7 ± 0.2
Large arterial compliance	mL/mmHg × 10	16.1 ± 0.8	14.8 ± 0.8	16.7 ± 1.1	16.3 ± 1.1

Data is presented as mean ± S.E.M. Abbreviations: FMD, flow-mediated vasodilation; PT, propagation time of the pulse wave from the time of ventricular ejection to the time that the pulse wave arrived at brachial/carotid artery; DC, distensibility coefficient

Other indicators of cardiovascular health, selected on scientific evidence that they may be affected by either wine or grape products, were also measured (Table 3). Platelet function, assessed by epinephrine/collagen or ADP/collagen-induced aggregation, was not changed and plasma lipid profiles were not affected. Plasma concentrations of the vasoconstrictor endothelin-1 were slightly decreased in the grape-wine intervention, and increased in the grape intervention.

**Table 3:** The effect of grape-wine extract and grape extract on platelet aggregation time and plasma markers.

Parameter	Units	Intervention group 1 (*n* = 30)		Intervention group 2 (*n* = 30)	
		Placebo	Grape-wine	Placebo	Grape
Epinephrine/collagen	s	114.7 ± 5.5	120.9 ± 5.5	129.6 ± 7.1	126.1 ± 6.5
ADP/collagen	s	84.1 ± 2.6	84.2 ± 2.6	86.9 ± 3.2	84.6 ± 3.0
Endothelin-1	pg/mL	1.55 ± 0.07	1.40 ± 0.07 ^†^	1.57 ± 0.05	1.70 ± 0.05 *
Total cholesterol	mM	5.81 ± 0.08	5.68 ± 0.08	5.94 ± 0.11	5.85 ± 0.11
LDL-cholesterol	mM	3.84 ± 0.08	3.74 ± 0.08	4.02 ± 0.09	3.91 ± 0.09
HDL-cholesterol	mM	1.60 ± 0.02	1.56 ± 0.02	1.57 ± 0.03	1.61 ± 0.03
Triglycerides	mM	1.34 ± 0.07	1.38 ± 0.07	1.26 ± 0.07	1.13 ± 0.07

Data is presented as mean ± S.E.M. ^†^ *p* = 0.05 and * *p* < 0.05 *versus* placebo. Platelet function data (aggregation time) is based on 25 subjects per intervention group.

Polyphenol profiling of the grape and wine extracts revealed that the wine contained (per gram): 21.5 mg anthocyanins, 45.2 mg catechins, 4.8 mg flavonols, 7.9 mg phenolic acids, and 1 mg stilbenes, whilst the grape contained 225.8 anthocyanins, 0.4 mg catechins, 9.5 mg flavonols, and 5.2 mg phenolic acids, as reported previously [14]. Although these data suggest that the wine extract is particularly rich in catechins and the grape extract rich in anthocyanins, one should realize that the majority of the polyphenols could not be identified, which were mainly polymeric proanthocyanidins. The specific polyphenols identified are listed in more detail in the supplementary research data, Table S2. Both grape-wine and grape intervention increased urinary excretion of a wide range of low-molecular weight phenolic acids (published previously) [14], the majority representing gut microbial degradation products of polyphenols.

## 5. Discussion

The present study demonstrates that consumption of a polyphenol-rich grape-wine extract containing 800 mg of polyphenols lowers SBP by 3 mmHg and DBP by 2 mmHg in untreated mildly hypertensive subjects. On an individual basis this may be a modest effect, but on a population-wide basis this would translate into a total risk reduction in excess of 10% for stroke and myocardial infarction [17]. In contrast, a grape juice extract did not affect BP. Because the amount of polyphenols consumed per day was the same in both interventions (800 mg), the results suggest that the red wine derived polyphenols, polyphenol classes and/or metabolites thereof may mediate the BP lowering effect. The grape juice is rich in anthocyanins and flavonols, while the red wine contributes particularly to ingestion of catechins, oligomeric procyanidins formed from catechins, and stilbenes, such as resveratrol [14]. The limited clinical studies with pure polyphenols indicate that epicatechin and epigallocatechin gallate (EGCG) may improve endothelial function [18,19]. These studies were, however, not optimally designed to explore the BP lowering effects of the compounds. In contrast, resveratrol has been tested on BP lowering efficacy and a recent meta-analysis suggests that it may exert BP lowering effects at very high concentrations (>150 mg/day), which is approximately 20-times higher than the levels of grape-wine extract in the current study [20]. Thus, catechins and procyanidins remain the most likely candidates that contribute to the vascular effects reported for red wine. This is supported by the growing evidence that consumption of cocoa, black tea and green tea lower blood pressure and improve vascular function, particularly endothelial function [21,22,23]. These polyphenol-rich food products are particularly rich in catechins or catechin-derivatives (procyanidins, theaflavins, thearubigins). Indeed, a systematic review by Kay *et al*. [24] concluded that catechins and procyanidins contribute to the vascular health benefits of flavonoids, and it is expected that clinical studies with pure single flavonoids will shed more light on the flavonoid structure-to-vascular benefit relationships [25].

A number of studies provide insight into the vascular effects of flavonoids. Indeed, Welch’s purple grape juice, high in anthocyanins and flavonols and low in catechins and procyanadins [26], was one of the first grape products with clinical evidence showing its consumption improved endothelial function [27,28]. Its effect on BP remained unclear [12], similar to our findings with the grape juice. Another trial in which a grape supplement containing high concentrations of flavans was consumed, including catechins, reported beneficial effects on BP and endothelial function [29]. Taken together, these studies suggest that a high concentration of catechins and procyanidins may be important if improvements in vascular health are to be achieved, particularly improvements in BP.

Both the trial design and the bioavailability of the study intervention are also important. Blood pressure lowering depends on the starting blood pressure of the study cohort with those with elevated blood pressure having more scope for lowering BP. Hence it is no surprise that previous studies in healthy, young normotensive subjects reported no change in BP following the ingestion of extracts of red wine and grape-wine alone [6,7]. Furthermore, 24 h-ambulatory BP measurements may offer more reproducible BP values due to the large number of readings and avoidance of white-coat hypertension. This method may therefore be more suitable to detect small changes in BP. This may explain why in the present study the BP effects with ambulatory BP assessment are more clear-cut than office BP measurements (the within-subject variance SD^2^ was 22 and 41 for 24 h-ambulatory BP and office BP, respectively). Bioavailability is a further consideration in the design of BP lowering trials studying polyphenol-rich products. In rats, the co-administration of quercetin (a flavonol) affected the bioavailability of (+)-catechin by extending the period of absorption [30]. In another study, Wang *et al*. [31] reported the uptake of EGCG by different cell types was actually enhanced when quercetin was co-administrated due to inhibition of catechol-o-methyl transferase (COMT) activity. This enhanced absorption was supported by data that showed EGCG accumulation in the kidneys and lungs of rats. These mechanistic studies therefore highlight the blood pressure lowering effect of catechins and procyanidins may depend on the presence of other (flavonoid) compounds in the foods being consumed.

The current study tested the impact of the polyphenol-rich extracts on nitric oxide (NO)-dependent vasodilation and circulating endothelin-1 (a vasoconstrictor). Results showed that the grape products tested had no effect on NO-mediated FMD of the brachial artery, which contradicts previous reports [32,33,34]. One explanation may be that maximal effects on FMD may have already been reached 30 min after ingestion [32], and the measurement 90 min later in this study cohort may have been suboptimal. In terms of endothelin-1, a significant reduction (−0.15 pg/mL) in plasma concentration was found in the grape-wine group. This finding complements previous *in vitro* studies showing that red wine decreases endothelin-1 plasma concentrations [34,35,36]. An unexpected finding was the increase in endothelin-1 plasma concentration in the grape juice intervention (+0.13 pg/mL).

Effects on platelet aggregation and plasma LDL-cholesterol, HDL-cholesterol and triglycerides concentrations were included in the current analysis. Table 3 shows that none of these parameters changed significantly (*p* > 0.05). This is in-keeping with the literature which reports inconsistent or negligible effects with polyphenol-rich foods [37] and red wine [38]. It is important to remember that in the current study the interventions contained no alcohol, which reportedly increases plasma HDL cholesterol [2,39].

Our study design had some limitations. The first relates to the four week intervention period, which was based on previous studies with polyphenol-rich products and may have been too short to detect meaningful changes. Indeed, pharmacological interventions take usually months to reach their maximal BP lowering effect. Therefore, the BP lowering effect of the grape-wine extract could be increased in a longer study. Placebo and grape product interventions were crossed-over after four weeks without a washout period, risking a carry-over effect. However, statistical analysis confirmed that no carry-over effect was seen for any of the parameters studied. BP lowering effects and the mechanism of action of the study extracts may have been more pronounced and definitive in hypertensive subjects with underlying vascular problems. On the other hand, patients receiving BP therapies may represent a heterogeneous group in which certain drugs would affect similar mechanisms as polyphenols, which would ultimately mask the effect of polyphenols. One improvement to the current study may have been the inclusion of a study arm testing the effect of red wine extract alone. The current study did not allow this due to logistical challenges.

This study makes a significant contribution to the scientific literature as it suggests that a range of catechins- and procyanidins-rich food products, including red wine, affects blood pressure and vascular health. It also suggests that a “magic bullet” for lowering BP may not exist and that efficacy is multifactorial, depending on the combination of flavonoid classes as well as the flavonoid (or metabolites of) bioavailability. Clearly more research is required to define the optimal flavonoid composition to achieve the optimal BP lowering effect.

## 6. Conclusions

Polyphenol-rich grape-wine extract lowers ambulatory systolic and diastolic BP, particularly during the daytime when blood pressure is higher, and this effect was independent of alcohol. Catechins and procyanidins are likely flavonoid classes contributing to this BP lowering effect. In the current study, the mechanism of action would seem to be explained by decreased plasma endothelin-1 concentrations as opposed to nitric oxide, which remained unchanged. The cardiovascular benefit of the grape-wine extract seems exclusively related to BP lowering and not to lipid metabolism or platelet function in healthy, mildly hypertensive subjects.

## Supplementary Files

Supplementary File 1
