# Supplementary material for: Consumption of a Polyphenol-Rich Grape-Wine Extract Lowers Ambulatory Blood Pressure in Mildly Hypertensive Subjects
Source: Nutrients. 2015 Apr 30;7(5):3138–53. doi: 10.3390/nu7053138 (PMC4446743; doi:10.3390/nu7053138)
Supplement: Supplementary File 1 [file nutrients-07-03138-s001.docx]

**Supplementary Research Data**

**Table S1.** Frequency of reported adverse events during the run-in and intervention periods.

| **Adverse event** | **Run-in**  **(*n* = 60)** | **Placebo**  **(*n* = 60)** | **Grape–wine**  **(*n* = 30)** | **Grape**  **(*n* = 30)** |
| --- | --- | --- | --- | --- |
| Acute bronchitis |  | 2 |  |  |
| Acute nasopharyngitis (common cold) | 5 | 7 | 1 | 2 |
| Adverse effects of work environment |  |  | 1 |  |
| Backache | 2 | 3 |  |  |
| Cardiac arrhythmia |  |  |  | 1 |
| Contusion elbow & forearm |  |  |  | 1 |
| Cough | 2 | 3 | 1 |  |
| Dental caries |  | 4 |  |  |
| Diarrhea |  |  |  | 1 |
| Dyspepsia |  |  | 1 |  |
| Epistaxis |  |  |  | 1 |
| Gastroenteritis |  | 3 |  |  |
| Headache | 2 | 6 | 2 | 1 |
| Influenza | 3 | 4 |  |  |
| Injury blood vessel |  |  | 1 |  |
| Myalgia | 2 | 3 |  |  |
| Nervousness |  |  | 1 |  |
| Periodontal and gingival disease |  | 2 |  | 1 |
| Premature heartbeats |  | 2 | 1 | 1 |
| Stenosis precerebral arteries |  |  |  | 1 |
| Unspecified arthropathy |  |  | 1 |  |

Most frequent reported adverse events (AEs) in alphabetical order noted during the run-in period
(*n* = 60 subjects) and after randomisation in one of the intervention groups (placebo 2 × 30 subjects, grape 30 subjects and grape-wine 30 subjects). AEs were not judged to be causally related to the test products. Frequency and number of AEs were too low to statistically associate specific AEs to
an intervention.

**Table S2.** Polyphenols identified in the grape-wine and grape extract.

| **Anthocyanins (mg/kg)** | **Grape-wine** | **Grape** |
| --- | --- | --- |
| delphinidin 3,5-diglucoside |  | 3,592 |
| cyanidin 3,5-diglucoside |  | 1,781 |
| delphinidin 3-glucoside | 471 | 3,227 |
| petunidin 3,5-diglucoside |  | 10,316 |
| cyanidin 3-glucoside | 110 | 1,294 |
| peonidin 3,5-diglucoside |  | 46,050 |
| malvidin 3,5-diglucoside |  | 82,753 |
| peonidin 3-glucoside | 1,757 | 5,402 |
| malvidin 3-glucoside | 9,263 | 11,655 |
| delphinidin 3-coumaroyl-5-diglucoside |  | 3,531 |
| cyanidin 3-coumaroyl-5-diglucoside |  | 933 |
| petunidin 3-coumaroyl-5-diglucoside |  | 7,332 |
| delphinidin 3-coumaroylglucoside | 352 | 1,091 |
| peonidin 3-coumaroyl-5-diglucoside |  | 5,373 |
| malvidin 3-coumaroyl-5-diglucoside |  | 34,509 |
| petunidin 3-coumaroylglucoside | 458 | 704 |
| peonidin 3-coumaroylglucoside | 795 | 647 |
| malvidin 3-coumaroylglucoside | 5,361 | 5,655 |
| petunidin 3-glucoside | 1,291 |  |
| peonidin 3-acetylglucoside | 153 |  |
| malvidin 3-acetylglucoside | 1,489 |  |
| **Sum** | **21,501** | **225,845** |
| **Catechins (mg/kg)** | **Grape-wine** | **Grape** |
| catechin | 12,845 | 134 |
| epicatechin | 12,170 | 95 |
| procyanidin B1 | 9,969 | 48 |
| procyanidin B2 | 6,777 | 15 |
| procyanidin C1 | 1,192 | 0 |
| gallocatechin | 618 | 49 |
| epigallocatechin | 201 |  |
| epicatechin-3-O-gallate | 1,407 | 57 |
| **Sum** | **45,178** | **398** |

**Table S2.** *Cont.*

| **Flavonols (mg/kg)** | **Grape-wine** | **Grape** |
| --- | --- | --- |
| hyperoside (quercetin-3-O-galactoside) | 130 | 101 |
| miquelianin (quercetin-3-O-glucuronide) | 1,779 | 2,486 |
| isoquercitrin (quercetin-3-O-glucoside) | 657 | 1,391 |
| qercitrin (quercetin-3-O-rhamnoside) + astragalin (kaempferol-3-O-glucoside) | 122 | 107 |
| quercetin | 466 | 1,170 |
| kaempferol | 50 | 161 |
| myricetin-3-O-glucoside | 941 | 2,448 |
| myricetin | 671 | 1,614 |
| **Sum** | **4,817** | **9,478** |
| **Phenolic acids (mg/kg)** | **Grape-wine** | **Grape** |
| caffeic acid | 1,268 | 299 |
| p-coumaric acid | 677 | 457 |
| ferulic acid | 71 | 90 |
| gallic acid | 1,464 | 2,015 |
| protocatechuic acid | 624 | 296 |
| p-hydroxybenzoic acid | 195 | 75 |
| vanillic acid | 394 | 191 |
| syringic acid | 1,019 | 996 |
| caftaric acid | 599 | 179 |
| coutaric acid | 785 | 92 |
| fertaric acid | 518 | 303 |
| ellagic acid | 237 | 193 |
| chlorogenic acid (5-O-caffeoylquinic acid) | 33 | 32 |
| **Sum** | **7,886** | **5,219** |
| **Stilbenes (mg/kg)** | **Grape-wine** | **Grape** |
| polydatin | 725 | 130 |
| trans-resveratrol | 244 | 20 |
| **Sum** | **969** | **150** |

© 2015 by the authors; licensee MDPI, Basel, Switzerland. This article is an open access article distributed under the terms and conditions of the Creative Commons Attribution license (http://creativecommons.org/licenses/by/4.0/).
